# Clinical value of artificial intelligence ultrasound-assisted diagnostic system in the differential diagnosis of benign and malignant thyroid nodules: A retrospective case-control study

**DOI:** 10.1097/MD.0000000000050126

**Published:** 2026-08-14

**Authors:** Meng Tian, Guangxi Liang, Xingwang Zhu, Yuetian Zhang, Haoyuan Zuo, Banggao Ni

**Affiliations:** a People’s Hospital of Deyang City, Endocrine Thyroid Disease Diagnosis and Treatment Center, Deyang, Sichuan, China; b Lanzhou University, The First School of Clinical Medical, Lanzhou, Gansu, China.

**Keywords:** artificial intelligence ultrasound, assisted diagnostic system (AI-UASD), benign thyroid nodules (BTNs), malignant thyroid nodules (MTNs)

## Abstract

Accurately distinguishing the benign thyroid nodules (BTNs) and malignant thyroid nodules (MTNs) is crucial for treatment planning and prognosis. This study aims to explore the clinical value of artificial intelligence ultrasound-assisted diagnostic system (AI-UADS) in diagnosing the BTNs and MTNs. In this retrospective case-control study, a total of 161 TNs from 91 patients confirmed by surgical pathology were included. All nodules underwent ultrasound examinations and were evaluated by both senior ultrasonographers and AI-UADS, independently. Using surgical pathology as the gold standard, the diagnostic performance of the 2 methods was analyzed using receiver operating characteristic curves (including classify the nodules based on size, location, capsule relationships, and presence or absence of Hashimoto thyroiditis [HT]). The area under the curve, accuracy, sensitivity, specificity, positive predictive value and negative predictive value of AI-UADS in the differential diagnosis of BTNs and MTNs were 0.933, 93.2%, 92.4%, 94.2%, 95.5%, and 90.3%, respectively, all of which were higher than ultrasonographers (0.821, 82.0%, 81.5%, 82.6%, 86.2%, and 77.0%, respectively) (all *P* < .05). Specifically, the accuracy in diagnosing nodules with a maximum diameter ≤10 mm (94.0% vs 80.7%) and in the left lobe (92.5% vs 81.3%); the accuracy, sensitivity, specificity, positive predictive value and negative predictive value in diagnosing nodules with far from the capsule ([93.8% vs 79.5%], [92.5% vs 75.5%], [94.9% vs 83.1%], [94.2% vs 80.0%], [93.3% vs 79.0%]) and without HT ([94.0% vs 82.7%], [93.2% vs 82.4%], [94.9% vs 83.1%], [95.8% vs 85.9%], [91.8% vs 79.0%]) of AI-UADS were higher than ultrasonographers (all *P* < .05). The AI-UADS demonstrates good diagnostic performance in differentiating BTNs and MTNs, and can serve as an effective auxiliary tool for ultrasonographers. It shows particular advantages in diagnosing nodules with a maximum diameter ≤10 mm, located in the left lobe, far from the capsule, and in patients without HT.

## 1. Introduction

Thyroid nodules (TNs) are common. Among them, benign asymptomatic nodules are recommended for observation, while malignant nodules are usually advised for surgical treatment.^[1]^ Therefore, accurately and effectively determining the nature of nodules is of vital importance for clinical treatment planning and outcome evaluation.^[2]^ At present, ultrasound examination is the most commonly used screening tool for TNs, which can stratify the risk of malignancy by analyzing the sonographic features of nodules using the Thyroid Imaging Reporting and Data System (TI-RADS).^[3]^ However, factors such as ultrasonographers’ experience, judgment, and attention can affect the accuracy of diagnostic results, leading to misdiagnosis or missed diagnosis. Different ultrasonographers may have significant differences in interpreting the same ultrasound image, increasing the uncertainty of diagnosis. In addition, manual diagnosis takes a long time and requires the participation of professionals, which increases the burden and cost to medical resources. To overcome the limitations of manual diagnosis, the introduction of artificial intelligence (AI) technology has become a key approach for improving the efficiency and accuracy of diagnosis.

Artificial intelligence ultrasound-assisted diagnostic system (AI-UADS) can automatically extract image features and classify them through algorithms such as deep learning, which is suitable for medical image analysis.^[4]^ At present, AI shows great potential in fields such as breast lesions and pulmonary nodules. Hao et al used machine learning methods, including logistic regression and decision trees to establish a quantitative analysis model, it can classify breast nodules as benign or malignant.^[5]^ AI also offers a promising supportive approach to lung cancer screening, presenting considerable potential in enhancing nodule detection sensitivity, reducing false‐positive rates, and classifying nodules.^[6]^ Several institutions worldwide have developed AI-UADS for TNs, which have been preliminarily applied in clinical practice.^[7–9]^ These studies have evaluated AI diagnostic performance by analyzing specific cases and comparing results with manual diagnosis, demonstrating that AI can reduce human error and improve diagnostic consistency. Notably, Peng et al conducted a large multicenter study involving 18,049 images from 8339 patients, demonstrating that deep learning-based AI models can effectively assist TN diagnosis and management.^[8]^ Swan et al performed external validation of the AIBx system for TN risk stratification, reporting improved sensitivity and specificity compared to TI-RADS.^[10]^ Wang et al specifically investigated dynamic AI ultrasound in patients with Hashimoto thyroiditis (HT), finding higher specificity and lower misdiagnosis rates than conventional ultrasound.^[11]^ Few studies have systematically compared AI-UADS versus ultrasonographers across multiple clinically relevant subgroups. These factors have direct implications for disease management: nodule size influences the threshold for biopsy and follow‑up intensity;^[12]^ anatomical location affects surgical approach and risk of extra-thyroidal extension;^[13]^ the nodule–capsule relationship is critical for assessing local invasiveness and planning the extent of resection;^[14,15]^ and HT status may alter the interpretation of nodules’ sonographic features.^[16]^ This study compared the diagnostic performance of AI-UADS and ultrasonographers in differentiating benign thyroid nodules (BTNs) from malignant thyroid nodules (MTNs), and deficiencies were addressed through detailed subgroup analyses: including nodule sizes (≤10, 10–20, and ≥20 mm), anatomical location (left lobe, isthmus, and right lobe), nodule–capsule relationship (capsule invasion, adjacent to capsule, and far from capsule), and HT status.

## 2. Participants and methods

### 2.1. Study participants

**Figure 1.**
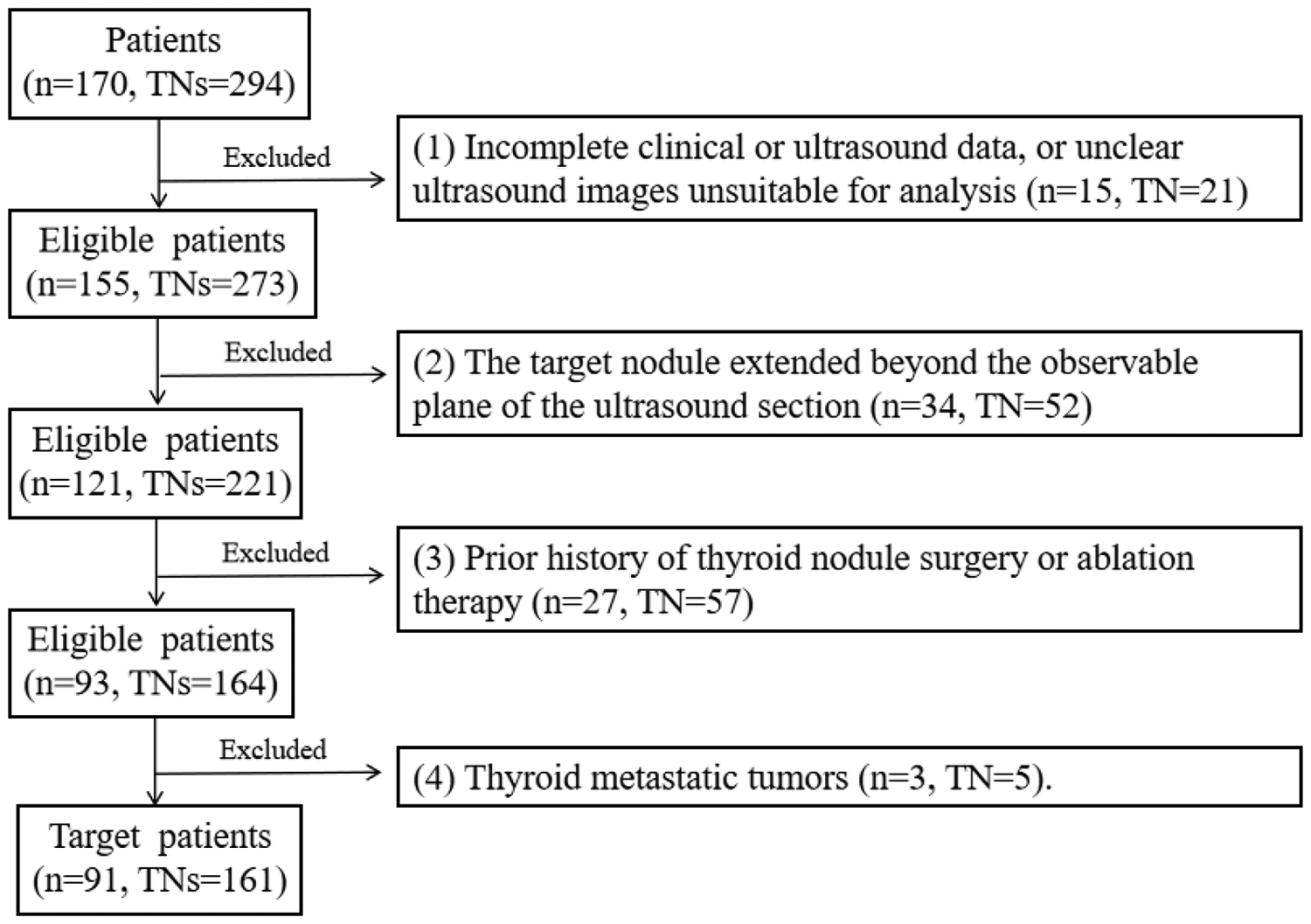
Flowchart of the inclusion and exclusion of patients (n = 91). TN = thyroid nodules.

A total of 91 patients with TNs confirmed by surgical pathology were selected between July 2024 and December 2025, which from People’s Hospital of Deyang City in Sichuan Province, China. The inclusion criteria: TNs initially diagnosed by ultrasound examination, the target nodule had definitive postoperative histopathological results, and standard transverse and longitudinal ultrasound images were retained. The exclusion criteria: Incomplete clinical or ultrasound data, or unclear ultrasound images unsuitable for analysis, the target nodule extended beyond the observable plane of the ultrasound section, prior history of thyroid nodule surgery or ablation therapy, and thyroid metastatic tumors (Fig. 1). This study was approved by the Ethics Committee of the People’s Hospital of Deyang City (ethical code: 2022-04-019-K01). As a retrospective study, patient informed consent was waived.

### 2.2. Methods

#### 2.2.1. Ultrasonographic Examination

Ultrasound examinations were performed using a Mindray R9 ultrasound diagnostic system (Mindray Medical Systems) equipped with a linear array probe (frequency: 4–18 MHz) set to the superficial organ mode. Patients were placed in the supine position with the neck region fully exposed. Using the preset thyroid examination protocol, the target nodule was positioned at the center of the image. The following features were observed: number, location, composition, echogenicity, shape, margin, calcifications, as well as the distance to the capsule and whether the capsule was smooth and intact. The maximum diameter of the nodule was measured on the longitudinal section. For patients with multiple nodules, the location and size of each nodule were marked during the examination. Both static and dynamic ultrasound images were saved. Three physicians, each with over 10 years of experience in thyroid-related ultrasonography, independently conducted a retrospective analysis of the patient’s ultrasound images. They scored and classified the nodules according to the 2017 version of the ACR TI-RADS diagnostic criteria,^[17,18]^ focusing on the primary indicators: composition, echogenicity, shape, margin, and calcifications. Any discrepancies were resolved through discussion.

#### 2.2.2. AI-UADS diagnosis

AI-UADS was performed by Ian Thyroid Solution 100 (ITS100) (MedAI Technology Co. Ltd). It is an ultrasonic image intelligent system, which is mainly composed of host, GE LOGIQ e ultrasonic diagnostic instrument, GE l4-12t-rs linear array probe and AI auxiliary display screen. AI-UADS utilizes computer vision and deep learning techniques, building an auxiliary diagnostic model for BTNs and MTNs based on a convolutional neural network (CNN). Important: the AI-UADS used in this study was a pretrained system with frozen model weights. Our dataset was completely independent and was not used in any phase of the AI system’s training, validation, or fine-tuning. The manufacturer (MedAI Technology Co., Ltd) confirmed that no data from our institution was included in their proprietary training corpus. Therefore, this study represents a fully external validation of the AI-UADS, with no risk of data leakage or overfitting. Before ultrasound image data put into the diagnostic model, a preprocessing module processes the images to obtain data that can be directly used by the model. Within the diagnostic model, deep learning technology is employed to automatically extract image features. Given the unique characteristics of ultrasound images, AI designed a novel CNN architecture for the diagnostic model, which samples pixels from the input images through CNN convolution to capture global image features of TN regions in ultrasound images. A high-throughput multi-level feature space is constructed, enhancing the diagnostic performance of the model. After extracting and computing features of the input nodule image, the diagnostic model outputs 2 probability values, indicating the likelihood that the input nodule is malignant or benign, respectively. When the predicted malignant probability is greater than or equal to the benign probability, the model’s prediction result is malignant, and a red “M” marker is displayed. Otherwise, the result is benign, and a green “B” marker is displayed. Percentages represent probabilities. The final AI diagnosis result is determined by majority vote: for each nodule, 3 separate ultrasound scans were performed, and each scan was independently analyzed by the AI system. The final AI diagnostic outcome was determined by majority vote among the 3 scan results. If at least 2 of the 3 scans were classified as malignant, the final diagnosis was recorded as malignant; if at least 2 scans were classified as benign, the final diagnosis was recorded as benign.^[19]^

#### 2.2.3. Subgroup analysis

First, size subgroup: based on the maximum diameter, nodules were categorized as ≤10, 10 to 20, and ≥20 mm.^[20]^ Second, location subgroup: based on the transverse plane, nodules were classified as located in the left lobe, isthmus, or right lobe. Third, nodule–capsule relationship subgroup: according to the distance from the nodule to the capsule and whether the capsule was smooth and intact, nodules were divided into capsule invasion (nodule < 2 mm from the capsule with interrupted capsular echo), adjacent to the capsule (nodule < 2 mm from the capsule with a continuous, smooth capsule), and far from the capsule (nodule ≥ 2 mm from the capsule).^[21]^ Fourth, HT subgroup: based on postoperative pathology, nodules were grouped into those with HT and those without HT. The accuracy, sensitivity, specificity, positive predictive value (PPV), and negative predictive value (NPV) of AI-UADS and ultrasonographers in differentiating BTNs and MTNs were compared across all subgroups.

### 2.3. Statistical analysis

Data management and analysis were performed using the SPSS 26.0 software (SPSS). The Shapiro–Wilk test was used to test the normality of the measurement data; normally distributed data were expressed as the mean ± standard deviation, and non-normally distributed data were expressed as the median (interquartile range), while qualitative categorical data variables were expressed as counts, percentages, and frequencies (rates). Intergroup comparisons were performed using the χ^2^ test or Fisher exact test. The consistency between AI-UADS and ultrasonographers was assessed using Kappa coefficient (κ). Using pathological results as the gold standard, receiver operating characteristic curves were plotted to evaluate the diagnostic performance of AI-UADS and ultrasonographers in differentiating BTNs and MTNs. The areas under the curve (AUC) were compared using the DeLong test. In all analyses, *P* < .05 was considered to be statistically significant.

## 3. Results

### 3.1. Demographic features of study participants

A total of 91 patients were included in this study, predominantly middle‑aged and older adults, with a mean age of 50.80 ± 11.07 years. Females significantly outnumbered males (75 vs 16), which is consistent with the known epidemiological pattern of TNs. At the nodule level, a total of 161 nodules were included, of which 92 (57.1%) were malignant and 69 (42.9%) were benign, indicating a relatively high proportion of malignant nodules. Multiple nodules (133) were far more common than solitary nodules (28), suggesting that most participants had multiple nodules. The median maximum diameter of the nodules was 10.0 (6.0–22.0) mm, indicating that the sample covered a range from small nodules to larger ones. This sample demonstrates good clinical representativeness and provides a reliable data foundation for subsequent comparisons of diagnostic performance between the AI-UADS and ultrasonographers across different subgroups (Table 1).

**Table 1:** Demographic features of study participants (n = 91).

Demographic features	
Age (yr)	50.80 ± 11.07
Height (cm)	158.95 ± 6.87
Weight (kg)	62.14 ± 11.17
Gender	
Male	16
Female	75
Thyroid nodules	
Solitary	28
Multiple	133
Malignant	92
Benign	69
Maximum diameter (mm)	10.0 (6.0, 22.0)

Age, height and weight are shown as mean ± SD; maximum diameter is shown as median (interquartile range).

SD = standard deviation.

### 3.2. The consistency analysis of diagnosis between AI-UADS and ultrasonographers

As shown in Table 2, the consistency analysis between the AI-UADS and ultrasonographers for predicting BTNs and MTNs showed a Kappa coefficient of 0.749 (*P* < .05). This indicates that the assessment results between the AI-UADS and ultrasonographers demonstrate good agreement. In other words, the AI-UADS demonstrated good agreement with ultrasonographers and could serve as a valuable adjunct tool to assist clinicians in decision-making.

**Table 2:** The consistency analysis of diagnosis between the artificial intelligence ultrasound-assisted diagnostic system and ultrasonographers.

Project	Kappa	*P*
Assessments	0.749	.000

### 3.3. Diagnostic findings of AI-UADS and ultrasonographers

**Figure 2.**
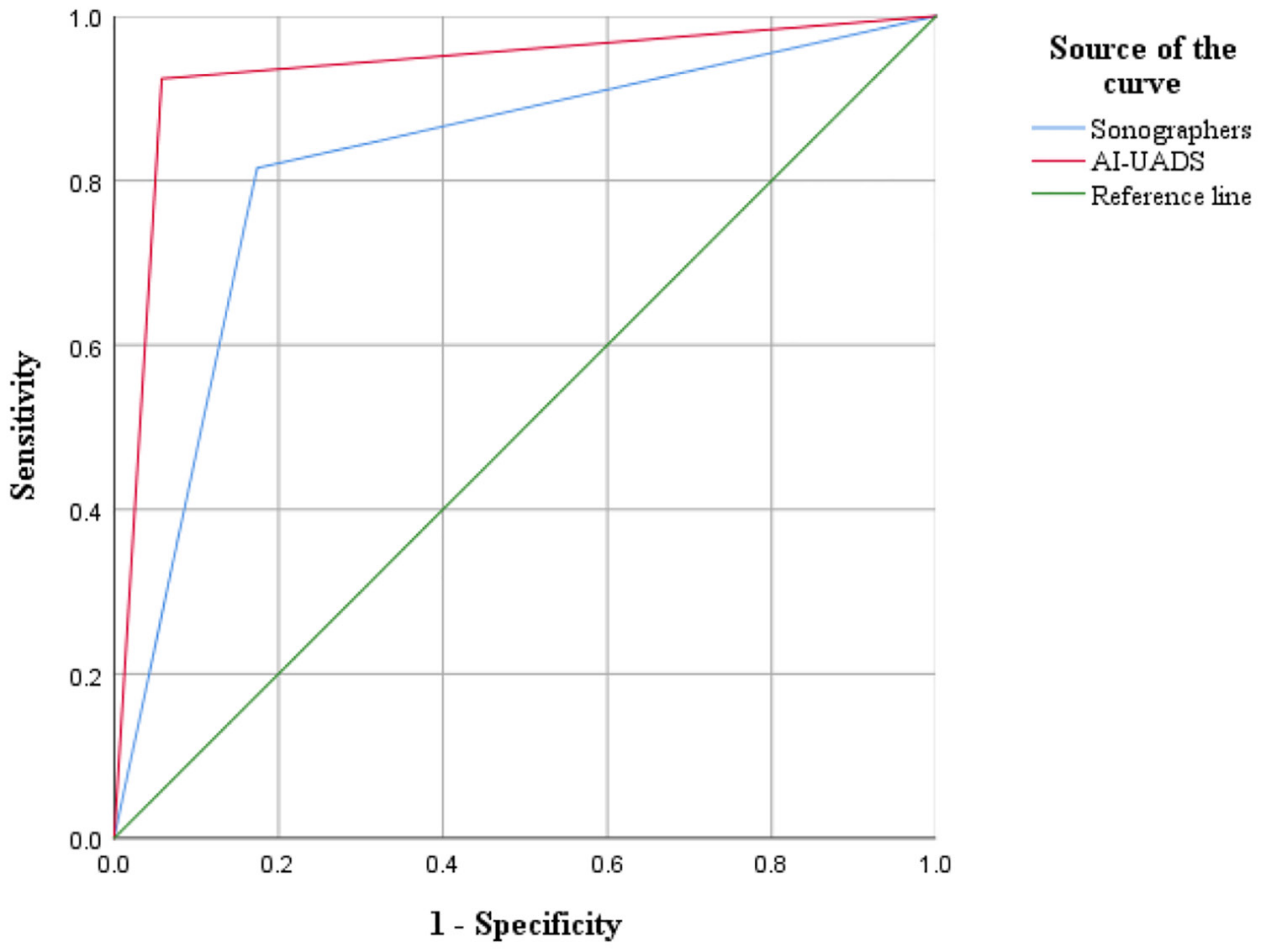
The ROC curves for the differentiation of BTNs and MTNs by AI-UADS and ultrasonographers. AI-UADS = artificial intelligence ultrasound-assisted diagnostic system, BTNs = benign thyroid nodules, MTNs = malignant thyroid nodules, ROC = receiver operating characteristic.

Among 161 TNs, pathology confirmed 92 MTNs and 69 BTNs. The ultrasonographers diagnosed 74 nodules as benign and 87 as malignant, accurately identifying 57 BTNs and 75 MTNs. AI-UADS diagnosed 72 nodules as benign and 89 as malignant, accurately identifying 65 BTNs and 85 MTNs. The receiver operating characteristic curve analysis (Fig. 2 and Table 3) showed that the AUC, accuracy, sensitivity, specificity, PPV, and NPV of AI-UADS in the differential diagnosis of BTNs and MTNs were 0.933 (95% confidence interval [CI], 0.888–0.978), 93.2% (95% CI, 88.9–96.3), 92.4% (95% CI, 87.1–96.0), 94.2% (95% CI, 88.4–97.6), 95.5% (95% CI, 90.5–98.3), and 90.3% (95% CI, 84.0–94.7), respectively, all of which were higher than those of ultrasonographers: 0.821 (95% CI, 0.751–0.890), 82.0% (95% CI, 75.9–87.2), 81.5% (95% CI, 74.6–87.3), 82.6% (95% CI, 74.7–88.9), 86.2% (95% CI, 79.4–91.5), and 77.0% (95% CI, 69.2–83.6). The differences were statistically significant (all *P* < .05).

**Table 3:** The diagnosis results of the artificial intelligence ultrasound-assisted diagnostic system and ultrasonographers were compared with the pathology (n = 161).

Pathological diagnosis	Diagnostic method				*X^2^*	*P*
	Ultrasonographers		AI-UADS			
	Malignant	Benign	Malignant	Benign		
Malignant (n = 92)	75	17	85	7	–	–
Benign (n = 69)	12	57	4	65		
Accuracy % (95% CI)	82.0 (75.9–87.2) (132/161)		93.2 (88.9–96.3) (150/161)		9.249	.002
Sensitivity % (95% CI)	81.5 (74.6–87.3) (75/92)		92.4 (87.1–96.0) (85/92)		4.792	.029
Specificity % (95% CI)	82.6 (74.7–88.9) (57/69)		94.2 (88.4–97.6) (65/69)		4.525	.033
PPV % (95% CI)	86.2 (79.4–91.5) (75/87)		95.5 (90.5–98.3) (85/89)		4.603	.032
NPV % (95% CI)	77.0 (69.2–83.6) (57/74)		90.3 (84.0–94.7) (65/72)		4.665	.031

AI-UADS = artificial intelligence ultrasound-assisted diagnostic system, CI = confidence interval, NPV = negative predictive value, PPV = positive predictive value.

### 3.4. Comparison of the diagnostic performance of AI-UADS and ultrasonographers in differentiating BTNs and MTNs in different subgroups

**Figure 3.**
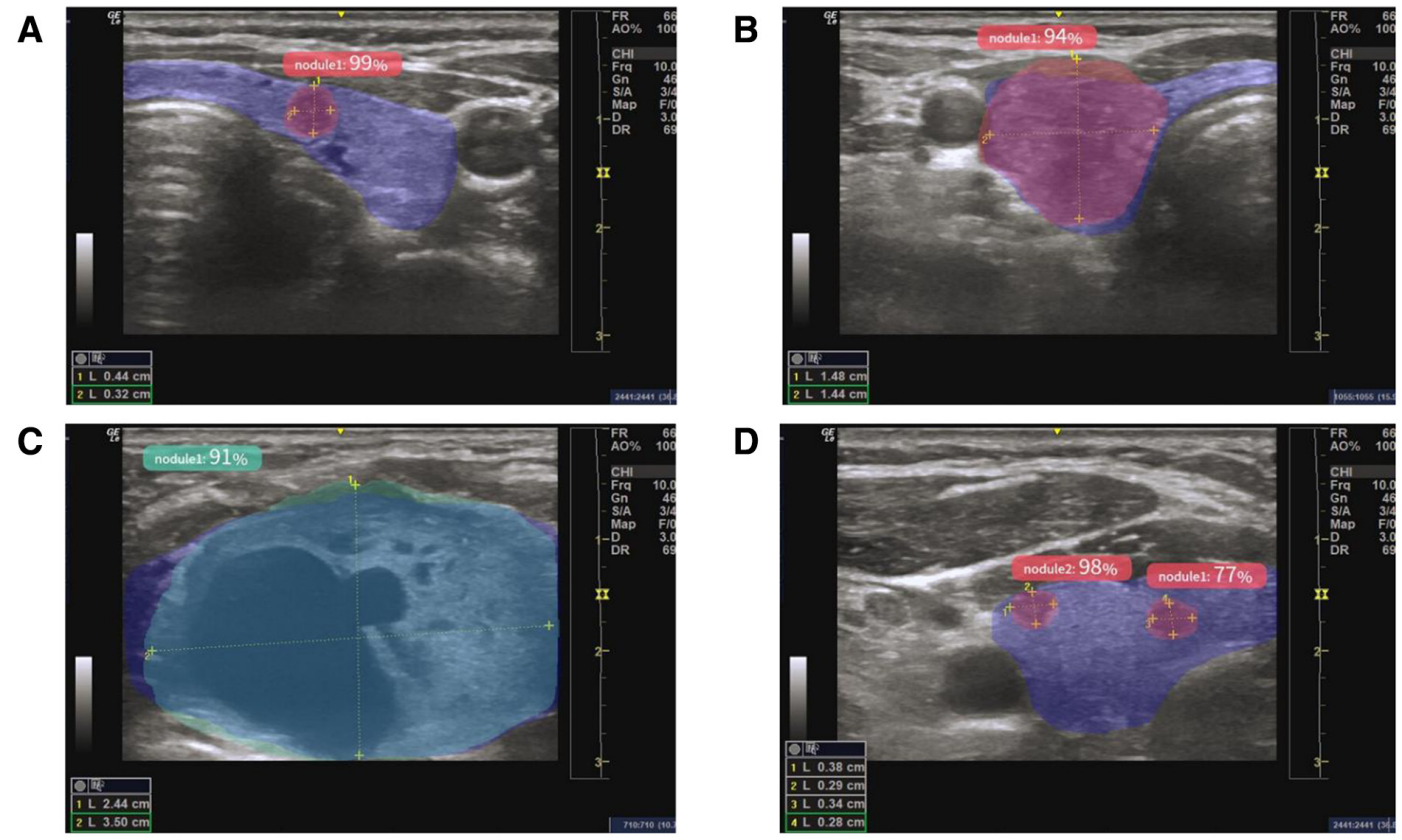
Ultrasound images of TNs. (A) A patient with papillary thyroid carcinoma (PTC) (female, 38 years). The nodule has a maximum diameter of 4.4 mm, is located in the right lobe near the isthmus, and is situated <2 mm from the capsule, which is smooth and intact. No HT was present. The ultrasonographer classified it as ACR TI-RADS 4b, while AI-UADS indicated a malignant probability of 99%. (B) A patient with PTC (female, 47 years). The nodule has a maximum diameter of 14.8 mm, is located in the left lobe, and is situated <2 mm from the capsule, which is interrupted. No HT was present. The ultrasonographer classified it as ACR TI-RADS 5, while AI-UADS indicated a malignant probability of 94%. (C) A patient with nodular goiter (female, 62 years). The nodule has a maximum diameter of 35.0 mm, is located in the right lobe, and is situated <2 mm from the capsule, which is smooth and intact. No HT was present. The ultrasonographer classified it as ACR TI-RADS 3, while AI-UADS indicated a benign probability of 91%. (D) A patient with PTC (female, 42 years). Nodule 1 has a maximum diameter of 3.8 mm, is located in the left lobe, and is situated <2 mm from the capsule, which is interrupted. HT was present. The ultrasonographer classified it as ACR TI-RADS 5, while AI-UADS indicated a malignant probability of 98%. Nodule 2 has a maximum diameter of 3.4 mm, is located in the left lobe, and is situated <2 mm from the capsule. The ultrasonographer classified it as ACR TI-RADS 4a, while AI-UADS indicated a malignant probability of 77%. AI-UADS = artificial intelligence ultrasound-assisted diagnostic system, HT = Hashimoto thyroiditis, PTC = papillary thyroid carcinoma, TNs = thyroid nodules, TI-RADS = Thyroid Imaging Reporting and Data System.

Comparison of the diagnostic performance between AI-UADS and ultrasonographers in differentiating BTNs and MTNs at different sizes: Among the 161 TNs, 83 had a maximum diameter of ≤10 mm (62 MTNs and 21 BTNs), 37 had a maximum diameter of 10 to 20 mm (20 MTNs and 17 BTNs), and 41 had a maximum diameter of ≥20 mm (10 MTNs and 31 BTNs). The accuracy of AI-UADS in differentiating BTNs and MTNs with a maximum diameter of ≤10 mm was higher than that of ultrasonographers (94.0% [95% CI, 86.7–97.4] vs 80.7% [95% CI, 70.9–87.8]) (*P* < .05). No significant differences were observed for nodules of 10 to 20 or ≥20 mm (Table 4 and Fig. 3A–D).

Comparison of the diagnostic performance of AI-UADS and ultrasonographers in differentiating BTNs and MTNs at different locations: among 161 TNs, 80 were located in the left lobe (40 MTNs and 40 BTNs), 4 were in the isthmus (2 MTNs and 2 BTNs), and 77 were in the right lobe (50 MTNs and 27 BTNs). The accuracy of AI-UADS in differentiating BTNs and MTNs in the left lobe was higher than that of ultrasonographers (92.5% [95% CI, 84.6–96.5] vs 81.3% [95% CI, 71.4–88.3]) (*P* < .05). No significant differences were found for nodules in the isthmus or right lobe (Table 4 and Fig. 3A–D).

Comparison of the diagnostic performance of AI-UADS and ultrasonographers in differentiating BTNs and MTNs with different capsule relationships: There were 112 nodules far from the capsule (53 MTNs and 59 BTNs), 14 nodules adjacent to the capsule (9 MTNs and 5 BTNs), and 35 nodules invading the capsule (30 MTNs and 5 BTNs). The accuracy, sensitivity, specificity, PPV, and NPV of AI-UADS in differentiating BTNs and MTNs far from the capsule were all higher than those of ultrasonographers (93.8% [95% CI, 87.7–97.0] vs 79.5% [95% CI, 71.1–85.9]), (92.5% [95% CI, 82.2–97.0] vs 75.5% [95% CI, 62.4–85.1]), (94.9% [95% CI, 86.1–98.3] vs 83.1% [95% CI, 71.6–90.6]), (94.2% [95% CI, 84.4–98.0] vs 80.0% [95% CI, 67.0–88.8]), (93.3% [95% CI, 84.1–97.4] vs 79.0% [95% CI, 67.4–87.4]) (all *P* < .05). No significant differences were observed for nodules adjacent to or invading the capsule (Table 4 and Fig. 3A–D).

Comparison of the diagnostic performance of AI-UADS and ultrasonographers in differentiating BTNs and MTNs with or without HT: Among the 161 TNs, 28 were associated with HT (18 MTNs and 10 BTNs), and 133 were not associated with HT (74 MTNs and 59 BTNs). For TNs not associated with HT, the accuracy, sensitivity, specificity, positive predictive value, and negative predictive value of AI-UADS in differentiating BTNs and MTNs were all higher than those of ultrasonographers (94.0% [95% CI, 88.6–96.9] vs 82.7% [95% CI, 75.4–88.2]), (93.2% [95% CI, 85.1–97.1] vs 82.4% [95% CI, 72.2–89.4]), (94.9% [95% CI, 86.1–98.3] vs 83.1% [95% CI, 71.6–90.6]), (95.8% [95% CI, 88.5–98.6] vs 85.9% [95% CI, 76.0–92.2]), (91.8% [95% CI, 82.2–96.5] vs 79.0% [95% CI, 67.4–87.4]) (all *P* < .05). No significant differences were found for nodules with HT (Table 4 and Fig. 3A–D).

## 4. Discussion

With the advancement of medical testing technology, the incidence of TNs has been increasing year by year. Traditional manual ultrasound diagnostic methods suffer from issues like strong subjectivity and low efficiency, highlighting the urgent need for more precise and efficient diagnostic approaches. AI-UADS builds a TN classification model based on deep learning algorithms, which are optimized and validated by integrating clinical medical knowledge to form a comprehensive diagnostic system. This study compared the diagnostic performance of AI-UADS and ultrasonographers in differentiating BTNs and MTNs under various clinical conditions (including stratification of nodule size, anatomical location variation, capsule relationships, and characteristics associated with HT). The findings indicate that AI-UADS significantly outperformed ultrasonographers across all diagnostic metrics, with higher AUC, accuracy, sensitivity, specificity, PPV, and NPV (all *P* < .05). Subgroup analyses revealed that AI-UADS was particularly effective for clinically challenging scenarios: small nodules (≤10 mm), nodules located in the left lobe, nodules far from the capsule, and nodules not associated with HT. In these subgroups, AI-UADS demonstrated significantly higher diagnostic accuracy compared to ultrasonographers (all *P* < .05). These findings suggest that AI-UADS can serve as a valuable auxiliary tool to enhance diagnostic precision in TN evaluation, particularly in specific clinical contexts where conventional ultrasound interpretation may face limitations.

Our study found that AI-UADS and ultrasonographers demonstrated good consistency in predicting the benign or malignant nature of TNs. Moreover, the AUC, accuracy, sensitivity, specificity, PPV, and NPV of AI-UADS are all higher than those of ultrasonographers, confirming the clinical application value of this standard. This may be attributed to several core advantages of AI-UADS. Firstly, AI-UADS enables quantitative feature extraction at the pixel level, such as the gray-scale value, echo distribution, morphological irregularity, and boundary gradient of nodules, whereas human clinicians typically rely on qualitative or semiquantitative descriptions like “high, medium, or low.” Secondly, the AI system was trained on a large dataset comprising hundreds of thousands of annotated images. This extensive training allows the system to identify feature combinations associated with malignancy that may escape human detection. Thirdly, the freedom from cognitive load and comprehensive scanning without omissions: In busy clinical settings, clinicians may have blind spots in attention or may be influenced by the most prominent features while overlooking other subtle signs. In contrast, AI systematically analyzes every region of the image, ensuring no learned suspicious features are missed. Previous studies have also confirmed this. For instance, Swan et al examined the performance of AI in the risk stratification of TNs and found that it minimized the subjectivity of ultrasound examinations.^[10,22]^ The outcomes indicated that when applied to US images of TNs, AIBx had better sensitivity, specificity, PPV, and a similar NPV when compared to the cancer risk stratification system TI-RADS.^[10]^ This offers us a “human-machine collaboration” model: AI performs initial screening and quantitative analysis, providing objective reference opinions, while ultrasonographers make the final judgment by integrating clinical information. This approach leverages the objective stability of AI while retaining the comprehensive decision-making ability of human clinicians. However, current studies have not thoroughly explored the specific aspects in which AI outperforms ultrasonographers. Therefore, this study conducted subgroup analyses, including nodule size stratification, anatomical location variations, capsule relationships, and characteristics associated with HT.

In clinical practice, subgroup analysis by size is widely applied in the diagnosis, treatment decision-making, and follow-up management of TNs, contributing to improved healthcare quality.^[23–25]^ This study found that AI-UADS demonstrated higher accuracy than ultrasonographers in differentiating BTNs and MTNs with a maximum diameter ≤10 mm, while its diagnostic efficacy for nodules >10 mm was comparable to that of ultrasonographers. This highlights the diagnostic advantages of AI in small TNs, offering certain clinical value and theoretical significance. Similar to our findings, the ACR TI-RADS shows better diagnostic efficacy on TNs >10 mm than on those ≤10 mm; it shows the low diagnostic efficacy of ACR TI-RADS on thyroid microcarcinoma.^[20]^ Two TNs with major diameters of 8 and 35 mm, both exhibiting strong hypoechogenicity and microcalcifications, are assessed as high risk regardless of their size disparity.^[26]^ However, certain ultrasound features of micro-nodules are often atypical, such as whether the margins are smooth and whether the shapes are regular. These key features are manifested very subtly on the tiny nodules, making it difficult for the human eye to distinguish them. This may also contribute to misdiagnoses by ultrasonographers.

The anatomical structures of the left lobe, isthmus, and right lobe of the thyroid gland are different, which leads to variations in the characteristics and implications of TNs. Multiple studies have shown that compared with other locations, nodules located in the isthmus and the upper and middle poles of the thyroid gland have a significantly higher risk of malignancy.^[27,28]^ Therefore, subgroup analysis based on the location of TNs contributes to a more accurate assessment of the condition. This study found that AI-UADS demonstrated higher accuracy than ultrasonographers in differentiating BTNs and MTNs located in the left lobe. In contrast, there were no statistically significant differences in accuracy, sensitivity, specificity, PPV, and NPV between AI-UADS and ultrasonographers for nodules in the isthmus or right lobe. The reasons for this location-specific difference in diagnostic performance are not entirely clear and require further investigation. Several hypotheses could be considered. Firstly, anatomical differences play a role: the adjacent structures of the left lobe are more “favorable.” The posteromedial aspect of the left thyroid lobe is adjacent to the esophagus, this serves as a clear and stable anatomical landmark.^[29]^ Physicians can easily locate the esophagus and use it as a reference to clearly observe the relationship between posterior left lobe nodules and the esophagus or trachea. In contrast, the adjacent structures of the right lobe are more “complex,” with major vessels such as the common carotid artery, internal jugular vein, and subclavian artery located posteriorly and laterally.^[30]^ The pulsation of these vessels can create motion artifacts, interfering with image stability. Secondly, the physiological habits of the operator: the majority of people around the world are right-handed, and ultrasonographers are no exception. When examining the patient’s left thyroid lobe, the ultrasonographers typically stands at the head or right side of the patient, with the right wrist in a natural and comfortable internally rotated position, allowing the probe to be held stably. When examining the right thyroid lobe while standing on the patient’s right side, the ultrasonographer’s right wrist must perform a significant outward rotation or adjustment. This subtle postural change may affect probe stability, particularly during detailed observations.^[31]^ However, these potential explanations remain speculative, as this study was not designed to investigate the mechanisms underlying location-based differences in diagnostic accuracy. Future studies specifically examining the relationship between nodule location, image characteristics, and the diagnostic performance of both AI and human readers would be valuable.

A large amount of data in clinical research and practice has shown that TNs with the characteristics of capsule invasion have a malignancy possibility of over 80-90%, or even higher.^[32,33]^Therefore, extra-thyroidal invasion is a critical assessment element in the ACR TI‑RADS classification, and its diagnosis has significant clinical significance. This study found that the accuracy, sensitivity, specificity, PPV, and NPV of AI-UADS in differentiating BTNs and MTNs far from the capsule were all higher than those of ultrasonographers, but there was no difference in invasive and adjacent to the capsule. This may be because “capsular invasion” itself is a clear malignant sign, enabling ultrasonographers to make accurate judgments based on this feature. In contrast, nodules that are “far from the capsule” lack such a direct clue, their benign‑malignant differentiation relies more on subtle analysis of complex internal features such as architecture, echogenicity, calcifications, and shape. This is precisely the domain where AI‑based image analysis may hold an advantage.

HT is significantly associated with the occurrence and development of thyroid cancer.^[34]^ Our research found that AI-UADS demonstrated good diagnostic performance in patients without HT, with no significant advantage over ultrasonographers in patients with HT, which contrasts with a previous study by Wang et al^[11]^ that reported that a dynamic AI system had significantly higher specificity and a lower misdiagnosis rate than preoperative ultrasound in patients with HT. HT is a chronic autoimmune disease in which the immune system attacks thyroid tissue.^[35,36]^ This impact on the overall thyroid tissue can significantly interfere with the ability of both ultrasonographers and AI-UADS to assess the nature of TNs, presenting a clinical challenge. Although our subgroup of patients with HT has a relatively small sample size, this does not affect the validity of our conclusions, as the AI-UADS had already undergone extensive pretraining prior to its deployment. This study merely serves as a validation of its performance, and thus, the conclusions are not influenced by the sample size. The discrepancies between our findings and those of Wang et al may be explained by certain methodological differences. Firstly, differences in the severity or sonographic phenotype of HT between the 2 study populations could influence AI performance, as the degree of parenchymal heterogeneity varies widely among patients. Secondly, although both systems utilize deep learning based on CNN, the specific architectures and training datasets likely differ. These comparisons highlight that future research should focus on multicenter cohorts of patients with HT, with careful stratification by the severity of sonographic changes. Furthermore, training AI models specifically on datasets enriched with HT cases, or developing specialized algorithms designed to disentangle nodule features from diffuse parenchymal abnormalities, may be necessary to fully realize the potential of AI-assisted diagnosis in this challenging patient population.

This study still has certain limitations. Firstly, the sample size was relatively small and derived from a single center. External validation requires larger sample sizes and further multicenter research to assess its impact on final clinical outcomes, such as the rate of unnecessary biopsies and treatment decisions. Secondly, this study was a retrospective analysis, which may introduce selection bias. In the future, prospective studies with larger sample sizes are needed to further evaluate the diagnostic efficacy between AI-UADS and ultrasonographers. Thirdly, several subgroups in our analysis had limited sample sizes (the isthmus location subgroup [n = 4]; the adjacent to capsule subgroup [n = 14]; the HT-positive subgroup [n = 28]). Future studies with larger, adequately powered cohorts are essential to confirm whether AI-UADS offers advantages in these specific clinical contexts.

## 5. Conclusions

In conclusion, AI‑UADS demonstrates high diagnostic performance in differentiating BTNs from MTNs, with significantly higher AUC, accuracy, sensitivity, specificity, PPV, and NPV than ultrasonographers. Its advantages are particularly pronounced for nodules ≤10 mm in maximum diameter, located in the left lobe, far from the capsule, and without HT. These findings confirm that AI-UADS can serve as an effective auxiliary tool to improve diagnostic precision, reduce operator dependency, and support clinical decision‑making in TN evaluation.

**Table 4-1:** Comparison of the diagnostic efficacy of the artificial intelligence ultrasound-assisted diagnostic system and ultrasonographers in differentiating benign thyroid nodules from malignant thyroid nodules in different subgroups (n = 161).

Nodule characteristics		Accuracy % (95% CI)				Sensitivity % (95% CI)				Specificity % (95% CI)			
		Ultrasonographers	AI-UADS	*X^2^*	*P*	Ultrasonographers	AI-UADS	*X^2^*	*P*	Ultrasonographers	AI-UADS	*X^2^*	*P*
Maximum diameter	≤10 mm	80.7 (70.9–87.8)	94.0 (86.7–97.4)	6.596	.010	85.5 (74.7–92.2)	95.2 (86.7–98.3)	3.321	.068	66.7 (45.4–82.8)	90.5 (71.1–97.4)	2.263	.133
	10–20 mm	75.7 (59.9–86.7)	89.2 (75.3–95.7)	2.333	.127	75.0 (53.1–88.9)	85.0 (64.0–94.8)	0.156	.693	76.5 (52.7–90.5)	94.1 (73.0–99.0)	–	.335
	≥20 mm	90.2 (77.5–96.1)	95.1 (83.9–98.7)	0.180	.672	70.0 (39.7–89.2)	90.0 (59.6–98.2)	–	.582	96.8 (83.8–99.4)	96.8 (83.8–99.4)	0.000	1.000
Locations	The left lobe	81.3 (71.4–88.3)	92.5 (84.6–96.5)	4.440	.035	77.5 (62.5–87.7)	92.5 (80.1–97.4)	3.529	.060	85.0 (70.9–92.9)	92.5 (80.1–97.4)	0.501	.479
	The isthmus	50.0 (15.0–85.0)	100.0 (51.0–100.0)	–	.429	100.0 (34.2–100.0)	100.0 (34.2–100.0)	–	–	0.0 (0.0–65.8)	100.0 (34.2–100.0)	–	.333
	The right lobe	84.4 (74.7–90.9)	93.5 (85.7–97.2)	3.240	.072	84.0 (71.5–91.7)	92.0 (81.2–96.9)	1.515	.218	85.2 (67.5–94.1)	96.3 (81.7–99.3)	0.882	.348
Capsule relationships	Far from the capsule	79.5 (71.1–85.9)	93.8 (87.7–97.0)	9.853	.002	75.5 (62.4–85.1)	92.5 (82.2–97.0)	5.675	.017	83.1 (71.6–90.6)	94.9 (86.1–98.3)	4.236	.040
	Adjacent to the capsule	78.6 (52.4–92.5)	92.9 (68.5–98.8)	–	.602	77.8 (45.3–93.7)	88.9 (56.5–98.0)	–	1.000	80.0 (37.6–96.4)	100.0 (56.6–100.0)	–	1.000
	Invading the capsule	91.4 (77.6–97.1)	91.4 (77.6–97.1)	0.000	1.000	93.3 (78.7–98.2)	93.3 (78.7–98.2)	0.000	1.000	80.0 (37.6–96.4)	80.0 (37.6–96.4)	0.000	1.000
HT	Yes	78.6 (60.5–89.8)	89.3 (72.8–96.3)	0.530	.467	77.8 (54.8–91.0)	88.9 (67.2–96.9)	–	.658	80.0 (49.0–94.3)	90.0 (59.6–98.2)	–	1.000
	No	82.7 (75.4–88.2)	94.0 (88.6–96.9)	8.216	.004	82.4 (72.2–89.4)	93.2 (85.1–97.1)	4.048	.044	83.1 (71.6–90.6)	94.9 (86.1–98.3)	4.236	.040

AI-UADS = artificial intelligence ultrasound-assisted diagnostic system, CI = confidence interval, HT = Hashimoto thyroiditis.

**Table 4-2:** Comparison of the diagnostic efficacy of the artificial intelligence ultrasound-assisted diagnostic system and ultrasonographers in differentiating benign thyroid nodules and malignant thyroid nodules in different subgroups (n = 161).

Nodule characteristics		PPV % (95% CI)				NPV % (95% CI)			
		Ultrasonographers	AI-UADS	*X^2^*	*P*	Ultrasonographers	AI-UADS	*X^2^*	*P*
Maximum diameter	≤10 mm	88.3 (77.8–94.2)	96.7 (88.8–99.1)	1.993	.153	60.9 (40.8–77.8)	86.4 (66.7–95.3)	3.737	.053
	10–20 mm	78.9 (56.7–91.5)	94.4 (74.3–99.0)	–	.340	72.2 (49.1–87.5)	84.2 (62.4–94.5)	–	.447
	≥20 mm	87.5 (52.9–97.8)	90.0 (59.6–98.2)	–	1.000	90.9 (76.4–96.9)	96.8 (83.8–99.4)	0.204	.651
Locations	The left lobe	83.8 (68.9–92.4)	92.5 (80.1–97.4)	0.696	.404	79.1 (64.8–88.6)	92.5 (80.1–97.4)	3.022	.082
	The isthmus	50.0 (15.0–85.0)	100.0 (34.2–100.0)	–	.467	0.0 (0.0–0.0)	100.0 (34.2–100.0)	–	–
	The right lobe	91.3 (79.7–96.6)	97.9 (88.9–99.6)	0.892	.345	74.2 (56.8–86.3)	86.7 (70.3–94.7)	1.501	.221
Capsule relationships	Far from the capsule	80.0 (67.0–88.8)	94.2 (84.4–98.0)	4.642	.031	79.0 (67.4–87.4)	93.3 (84.1–97.4)	5.200	.023
	Adjacent to the capsule	87.5 (52.9–97.8)	100.0 (67.6–100.0)	–	1.000	66.7 (30.0–90.3)	83.3 (43.7–97.0)	–	1.000
	Invading the capsule	96.6 (82.8–99.4)	96.6 (82.8–99.4)	0.000	1.000	66.7 (30.0–90.3)	66.7 (30.0–90.3)	0.000	1.000
HT	Yes	87.5 (64.0–96.5)	94.1 (73.0–99.0)	–	.601	66.7 (39.1–86.2)	81.8 (52.3–94.9)	–	.640
	No	85.9 (76.0–92.2)	95.8 (88.5–98.6)	4.255	.039	79.0 (67.4–87.4)	91.8 (82.2–96.5)	4.014	.045

AI-UADS = artificial intelligence ultrasound-assisted diagnostic system, CI = confidence interval, HT = Hashimoto thyroiditis, NPV = Negative predictive value, PPV = Positive predictive value.

## Acknowledgments

We thank MedAI Technology Co. Ltd, Wuxi, China, for their instruction and assistance. We thank PaperPal Preflight for English language editing.

## Author contributions

**Conceptualization:** Guangxi Liang, Banggao Ni.

**Data curation:** Meng Tian, Xingwang Zhu.

**Formal analysis:** Meng Tian.

**Investigation:** Guangxi Liang.

**Methodology:** Meng Tian, Yuetian Zhang, Haoyuan Zuo.

**Project administration:** Banggao Ni.

**Software:** Yuetian Zhang, Haoyuan Zuo.

**Supervision:** Banggao Ni.

**Validation:** Meng Tian.

**Writing – original draft:** Meng Tian.

**Writing – review & editing:** Guangxi Liang, Xingwang Zhu.
